# A mixed-method study to improve outcome of mass drug administration in two TAS failed districts of Uttar Pradesh, India

**DOI:** 10.1371/journal.pntd.0013112

**Published:** 2025-07-07

**Authors:** Achintya Srivatsa, Padmalochan Biswal, Satyabrata Routray, Shweta Prasad, Kevin Bardosh, Paresh Kumar, Amresh Kumar, Aurpit Patnaik, Shoaib Anwar, Vindu Prakash Singh

**Affiliations:** 1 Infectious Diseases, PATH, New Delhi, India; 2 Department of Sociology, Banaras Hindu University, Varanasi, India; 3 Department of Global Health, University of Washington, Seattle, Washington, United States of America; 4 Public Health, DevInsights, Noida, India; 5 Infectious Diseases/ Neglected Tropical Diseases, PATH, Lucknow, India; 6 Vector Borne Diseases, Government of Uttar Pradesh, Lucknow, India; Raiganj University, INDIA

## Abstract

Lymphatic filariasis (LF), commonly called “elephantiasis,” is one of the leading causes of disability around the world, with approximately 863 million people across 47 countries still living under threat of LF infection. India, with 40% of the global LF burden, is the most affected country that undertakes annual mass drug administration (MDA) using double and triple drug chemoprophylaxis in 157 LF-endemic districts. Consumption of drugs during MDA campaigns remains the biggest challenge for disease elimination in the country. PATH conducted a dual-phased study titled, “A Mixed-Method Study to Improve outcome of Mass Drug Administration in Two TAS failed Districts of Uttar Pradesh, India.” The study aimed to identify the critical factors in achieving optimal drug consumption among community members during MDA campaigns (pre-intervention phase), develop an intervention package (IP) for use in the government's subsequent MDA program to improve the coverage, and assess the impact (post-intervention phase). The quantitative data collection entailed house-to-house and drug administrator surveys, while qualitative data collection included in-depth interviews and rapid ethnography. The main reason for non-consumption was found to be fear of side effects, followed by away from home, lack of awareness, and suboptimal micro-planning. Post-IP measurements showed that there was an increase in evaluated coverage of 10.1% in Varanasi and 20.7% in Chitrakoot, as well as an increase in community awareness of 40% in Chitrakoot, with interpersonal communication being the most effective method for demand generation/ community awareness. An Intervention Package (IP), developed and piloted in the two study districts, was adopted by the state government of Uttar Pradesh and used across all 50 LF-endemic districts in the state.

## Author summary

Lymphatic filariasis (LF), a debilitating mosquito-borne disease, continues to affect millions globally, with India bearing the highest burden. Mass Drug Administration (MDA) is a key public health strategy to eliminate LF, but many districts in Uttar Pradesh have struggled with low drug consumption rates despite repeated MDA rounds. This mixed-method study addresses that challenge by examining why people don't take the LF drugs and testing a tailored intervention package (IP) in two under performing districts, Varanasi and Chitrakoot.

Using household surveys, interviews, and ethnographic methods, the study identified critical barriers to drug uptake—fear of side effects, lack of awareness, and mistrust in health workers. Based on these insights, a targeted IP was developed and piloted. Post-intervention, both districts showed marked improvements in drug coverage and community awareness, with Chitrakoot’s awareness levels nearly doubling.

This work is important because it offers an evidence-based model for improving MDA outcomes through community-centered and system-level changes. For scientists, it contributes to implementation science in disease elimination. For broader audiences, it highlights how tailored, empathetic public health strategies—grounded in local realities—can accelerate progress against neglected tropical diseases and improve health equity in underserved populations.

## Introduction

Lymphatic filariasis (LF), commonly called “elephantiasis,” is one of the leading causes of disability around the world including India. Lymphatic Filariasis (LF) is a neglected tropical disease caused by Filarial parasites, primarily Wuchereria bancrofti, Brugia malayi, and Brugia timori. It spreads through mosquito bites, particularly from Culex species.

By addressing the root causes and implementing preventive strategies, LF can be effectively controlled and eliminated. These are;

- **Mass Drug Administration (MDA)** – WHO recommends periodic administration of antifilarial drugs (*Ivermectin, Albendazole, and Diethylcarbamazine*) in endemic areas.
- **Mosquito Control** – Use insecticide-treated nets (ITNs), indoor residual spraying, and larval source management to reduce mosquito populations.
- **Improved Sanitation** – Proper waste disposal and drainage management to eliminate mosquito breeding sites.
- **Personal Protection** – Wearing long-sleeved clothing and using mosquito repellents can reduce mosquito bites.
- **Health Education** – Raising awareness in affected communities about transmission, symptoms, and preventive measures.
- **Early Diagnosis and Treatment** – Prompt detection and treatment of infections can prevent disease progression and disability

### Mass drug administration (MDA)

Mass Drug Administration (MDA) is a public health strategy recommended by the World Health Organization (WHO) to eliminate Lymphatic Filariasis (LF) in endemic regions. The goal is to interrupt transmission by administering antifilarial drugs to entire at-risk populations, regardless of infection status [1].

1. **Drugs Used in MDA in India**

The following combination of drugs are used in India:

-  Diethylcarbamazine (DEC) + Albendazole (ALB)
-  Triple Therapy: Ivermectin (IVM) + DEC + Albendazole (ALB) – A newer WHO-recommended regimen for rapid LF elimination being used in selected districts since 2019 [2].

2. **Mode of Transmission**

LF is transmitted through the bite of infected mosquitoes When an infected mosquito bites a person, filarial larvae (microfilariae) enter the bloodstream and develop into adult worms in the lymphatic system, causing blockages and complications such as elephantiasis and hydrocele.

3. **Side Effects of MDA Drugs**

MDA drugs are generally safe, but some individuals may experience mild to moderate side effects due to the immune system reacting to the dying parasites. These drugs are consumed orally. Common side effects include:

-  Mild reactions: Headache, dizziness, nausea, fever, fatigue.
-  Moderate reactions: Vomiting, diarrhea, muscle pain, skin rashes.
-  Severe reactions (rare): Allergic reactions or worsening symptoms in people with high microfilariae loads.

These side effects are temporary and usually subside within a few days.

4. **Storage and Transportation Temperature**

To maintain drug effectiveness, proper storage and transportation conditions must be followed:

-  Diethylcarbamazine (DEC): Store below 30°C, away from moisture and direct sunlight.
-  Albendazole (ALB): Store at 15°C – 30°C, in a dry place.
-  Ivermectin (IVM): Store at 15°C – 25°C, protected from light and moisture.

During transportation, drugs should be kept in temperature-controlled environments to prevent degradation, ensuring their efficacy during MDA campaigns.

In India, the National Programme to Eliminate Lymphatic Filariasis was launched in 1996/1997 on a pilot scale covering 13 lymphatic filariasis (LF)–endemic districts in seven states. In 2004 the government of India launched it on a national scale and began a nationwide mass drug administration (MDA), with the aim of eliminating LF as a public health problem [3].

Transmission Assessment Survey (TAS) is a critical tool used to determine whether lymphatic filariasis (LF) transmission has been reduced to levels low enough to stop mass drug administration (MDA) campaigns. TAS evaluates the presence of LF infection in children, who serve as sentinel indicators for ongoing transmission. If the survey results show that infection rates are below a certain threshold (Microfilariae rate of <1), it indicates that transmission is no longer sustainable in the population, and MDA campaigns can be safely halted.

At present, of the 256 LF-endemic districts, 99 have stopped MDA after passing the Transmission Assessment Survey (TAS) and are under post-MDA surveillance. The remaining 157 districts are either implementing MDA or preparing for the TAS. Uttar Pradesh (UP) is one of the high-focus states, as it accounts for about one-third (51) of the 157 districts with persistent transmission of LF. During 2016, 18 districts of UP conducted the TAS, and 14 of them (78 percent) failed to pass the first TAS. The reported MDA coverage in UP has always remained very high, but the assessed coverage has always demonstrated suboptimal compliance for drug consumption during MDA campaigns. It has been observed that, even with a high reported MDA coverage and microfilaria rates of < 1%, districts in UP have failed to clear the TAS [4].

## Method

### Ethical considerations

Approval for the study was received internationally from the Western Institutional Review Board, or WIRB (now Western-Copernicus Group IRB - WCG IRB), and locally from Sigma-IRB, New Delhi, India. Written consent was obtained from all individuals who participated in the study.

### Methodology

The study was conducted in the districts of Varanasi and Chitrakoot in Uttar Pradesh. These two districts were selected for the study because they had failed their respective transmission assessment surveys, have suboptimal health indicators, and are geographically and demographically representative of urban as well as rural settings with endemic lymphatic filariasis (LF) district in the country.

### Geographical situation of study districts

**Varanasi** - Varanasi district is located in the eastern part of Uttar Pradesh, India, covering an area of 1,535 km^2^. It lies between 25.0°N to 25.5°N latitude and 82.5°E to 83.0°E longitude, with an average elevation of 80 meters above sea level. The Ganges River flows through the district, making the land highly fertile. According to the 2011 Census, Varanasi district had a population of approximately 3.68 million people. The population density is around 2,400 people per km^2^. The literacy rate is about 77%, higher than the national average. The district has a sex ratio of around 909 females per 1,000 males. Varanasi is one of the oldest cities in the world.

**Chitrakoot** - Chitrakoot district is located in the Bundelkhand region of Uttar Pradesh, sharing its border with Madhya Pradesh. It lies between 24.48°N to 25.25°N latitude and 80.58°E to 81.34°E longitude, covering an area of 3,216 km^2^. The district is characterized by hills, plateaus, and forests, with the Vindhya Mountain range passing through it. The climate is semi-arid, with hot summers, moderate monsoons, and cool winters. According to the 2011 Census, Chitrakoot district had a population of approximately 990,626 people, with a population density of 308 people per km^2^. The literacy rate is around 66%, lower than the national average. The sex ratio stands at about 879 females per 1,000 males. The district has a predominantly rural population, with agriculture as the main occupation.

The study had two phases—the pre-intervention (baseline) and post-intervention (end-line) phases. In the pre-intervention phase, baseline data were collected to establish coverage and compliance benchmarks and to understand factors that hindered uptake of LF drugs among community members. Findings from the baseline survey helped in the development of an intervention package, used by the government in the subsequent MDA campaign. After the campaign, a post-implementation study was conducted to assess the impact of the intervention package on reach and consumption of LF drugs during MDA rounds.

The study used a mixed-methods approach consisting of three methods: quantitative, qualitative, and rapid ethnography. This was an explanatory sequential design type mixed method study where the quantitative data was initially collected from the community regarding MDA coverage and the DA and IDIs & rapid ethnography was done to explore the possible reasons behind the low coverage of the MDA campaign. The quantitative data was used to measure key quantifiable indicators like LF awareness and drug compliance. The qualitative component provided deeper understanding about the knowledge base and barriers to accessing LF drugs and complying with the treatment, the prevalence of LF in the villages, etc. Rapid ethnography was used because of its ability to generate rich and in-depth data about the contextual barriers and drivers in the two districts, including a wide range of stakeholder perspectives, and to explore reasons for coverage and compliance of LF drugs, through a human-centric approach. An Intervention Package (IP) was developed after the pre-intervention phase based on the analyzed data to improve the MDA campaign The IP was implemented in the subsequent MDA campaign in the study districts and In the post-intervention phase, the effectiveness of the IP was measured in terms of improvement in the MDA coverage.

The study was initiated in August 2020, when the data collection for the pre-intervention phase started and was completed in October 2020. This was followed by 2 months of data analysis and a further 2 months of development of the IP. The IP was utilized in August 2020 MDA round (which had got delayed due to ongoing COVID19 Pandemic) and the measurement of IP was done in September-October 2021. Table 1 gives the summary of the study types, method and the study population.

**Table 1:** Summary of study types, methods, and population.

Study type	Method	Study population
Quantitative	Household survey	Household members
	Drug administrator survey	Drug administrators
Qualitative	In-depth interviews	Community leaders and influencers Block- and district-level officers
	Ethnography	Those who consume drugs during MDA and those who do not Traditional healers Community health workers

### Study participant and sample size

The study followed a cluster survey design to enroll the required number of participants. Required sample size for estimating an increase of 10% in the assessed coverage over time had been derived using the following steps, in which P1 = estimated MDA coverage at the baseline, P2 = estimated MDA coverage at the end-line (P1 + 10%), P = pooled mean (P1 + P2)/2, α = probability of a Type 1 error, and β = power of the test [4]:

- **Step 1**. Estimation of effective sample size, which was calculated using the following formula

$$n^{\prime }\geq \left\{{[z_{\left\{1-\alpha \right\}\sqrt{\left\{2\overset{―}{\left\{P\right\}\left(1-\overset{―}{\left\{P\right\}}\right)}\right\}}}+z_{\left\{1-\beta \right\}\sqrt{\left\{P_{1\left(1-P_{1}\right)}+P_{2\left(1-P_{2}\right)}\right\}}}]}^{2}\right\}/\left\{{\left(P_{2}-P_{1}\right)}^{2}\right\}$$

Equation 1 - **Estimation of effective sample size**

Assuming P1 as 50% and P2 as 60% with 95% level of confidence and 80% power, resulting in 385 as the effective sample size for detecting required change over time.

- **Step 2**. Continuity correction, in which, after application of the continuity correction with the desired significance level and power, the required effective sample size had been computed as 404.7 (405) using the following formula:

$$n\geq \left\{n^{\prime }\right\}/\left\{4\right\}{[1+4/\left\{n^{\prime }\left|P_{2}-P_{1}\right|\right\}\;]}^{2}$$

Equation 2 - **Continuity correction**

- **Step 3**: Measure of expected impact, considering a design effect of 1.32 (rural UP National Family Health Survey 4), with the required sample size for the desired change equaling 534 (405 X 1.32).

Using the above assumptions and based on a two-tailed sample size calculation [5], we calculated that a minimum of 30 clusters (at 19 households, or HHs, per cluster) are needed to detect a 10-percentage point increase in assessed MDA coverage. This comes to a total of 570 HHs at both baseline and end-line. Table 2 below illustrates the study participants and required sample size for the study and the actual data collection nos.

**Table 2:** Sample size, per district.

*District*	*Total cluster*	*Interview per cluster (Households)*	*Target Sample Size (DA)*	*Actual DA interviewed in 1* ^*st*^ * phase*	*Actual DA interviewed in 2* ^*nd*^ * phase*	*Targeted* *Sample Size (HH)*	*Actual HH Visited in 1* ^*st*^ * phase*	*Actual HH Visited in 2* ^*nd*^ * phase*
*Varanasi*	*30*	*19*	*30*	*30*	*30*	*570*	*663*	*663*
*Chitrakoot*	*30*	*19*	*30*	*30*	*30*	*570*	*658*	*658*

A total of 30 clusters in both phases were covered. The clusters (village/wards) were selected by using the probability proportionate to size method with data from the 2011 census. From each study cluster, 19 HHs were selected through systematic random sampling.

For qualitative data collection, purposive sampling was employed. The objective was to understand the enablers and barriers of the MDA program, so personnel with several years of experience in MDA campaigns were interviewed. Additionally, community leaders residing in the clusters where HtH surveys were conducted were also interviewed. The rapid ethnography was conducted in six villages (three in each study district).

### Quantitative data collection

#### HH surveys.

The HH surveys were conducted to compare the assessed coverage data of two MDA rounds, the first before implementation of the IP and the second after implementation of the IP. In addition, the HH surveys also captured data on enablers and barriers taking the communities perspective.

#### Drug administrator (DA) surveys.

DA surveys were done to find out enablers and barriers with respect to DA’s perspective. The survey was conducted in pre- and post-intervention phases. A semi-structured questionnaire was used for the survey to understand the relationship between DAs’ socioeconomic/job-related factors, attitudes, performance, retention, and professional quality of life. Additionally, a motivation score was calculated based on the sum of the responses (up to 20 on the Likert scale), with questions ranging from 1 (strongly disagree) through 5 (strongly agree).

### Qualitative data collection

#### In-depth interviews (IDIs).

IDIs were conducted in both pre- and post-intervention phases. The IDIs were done for community leaders and health officials at block and district levels. The pre-intervention phase IDIs helped gather information on community perception of LF elimination, MDA campaigns, and the strengths and weaknesses of the health system.

#### Rapid ethnography.

Rapid ethnography is a qualitative research method that emphasizes quick, immersive engagement with participants in their natural environments. This approach allows researchers to observe behaviors, interactions, and cultural contexts in real time, providing a deeper understanding of the subjects experiences and perspectives. By prioritizing direct interaction and observation, rapid ethnography captures nuanced insights that traditional research methods may overlook, making it particularly effective for exploring complex social issues.

Rapid ethnography was done only in the pre-intervention phase. Three villages were chosen from each of the two study districts, and then mapping exercises, key informant interviews, case interviews, mini-interviews, transect walks, and short surveys were conducted. The study relied on a mixture of snowball, opportunistic, purposive, and maximum-variation sampling. Table 3 summarizes the rapid ethnography framework and the sampling breakdown, respectively.

**Table 3:** Rapid ethnography framework.

Type of sample	Type of exercise
Flexible sample frame determined by each team	Transect walks Informal group discussions Mini interviews Participant observation Review of health promotion material
Quota sample frame determined by the supervisor	Social mapping Key informant interviews with formal/informal leaders and health workers/volunteers Case interviews (half compliant with MDA/half not compliant)

### Training

The field team were trained before both phases and were oriented on the National Programme for Elimination of Lymphatic Filariasis (i.e., the objectives and implementation of the program, along with its issues and challenges) and use of each of the study instruments, as well as the critical aspects of the collection of high-quality data, observation techniques, and how to verify and scrutinize the data.

Ethnographers were oriented and trained on the goals of the study as well as on the methodological approach. The training session was built upon the explanation on the research tools that were prescribed for this study and the nuances of anthropological fieldwork and ethnographic studies.

### Data analysis

#### Quantitative.

Data cleaning, descriptive statistics, and finding statistically significant associations and differences were the three main components of quantitative data analysis including Descriptive statistical analysis. Since most of the variables were nominal, a chi-squared test was used to see whether a statistically significant difference occurred between key study indicators across pre- and post-implementation phase.

#### Qualitative.

While IDIs with community leaders and influencers provided critical insights into some of the demand-side barriers to MDA compliance, interactions with government officials helped in identifying some of the supply-side barriers. The qualitative data collected were first transcribed and translated into English. After transcription, a detailed coding framework was developed according to various themes that emerged—such as distribution of medicines, capacity-building, logistics, etc.—that served as a reference while analyzing the qualitative data. Deductive thematic analysis was utilized wherein the researchers identified themes such as distribution of medicines, capacity building, logistics, etc, which they expected to find in the data. These themes were derived from the research objectives and the conceptual framework guiding the study and from literature review and past experiences on working in the NTDs. The aim was to see how well these predefined themes are represented in the data or to understand the data in the context of these frameworks.

The deductive coding process involved the following key steps:

-  First, researchers familiarized themselves with the data through repeated readings, defining theoretical frameworks, conducting an in-depth review of the transcripts and research questions.
-  Next, they applied the predetermined coding framework to the data, coding segments of text according to the predefined themes. As the coding process progressed, the pre-determined codes were continually refined, and sub-themes were developed as needed to capture nuances in the data.
-  Finally, the coded data was reviewed and organized to ensure they align with the thematic framework, to refine or consolidate key themes as necessary.

The inter-coder reliability exercise was not done during the qualitative analysis.

Continuous iterative analysis was done to data during collection to identify emerging themes and gaps. Stopping Criteria was set when a specific number of consecutive interviews without new themes as a saturation point.

For rapid ethnography, thematic data analysis was carried out in a group setting with all the ethnographers, facilitated by a senior scientist. The thematic coding approach used during the group analysis involved using an alphabetical and color-based system.

### Intervention package (IP)

Analysis of the quantitative and qualitative data of the pre-intervention phase of the study was used for development of the IP. This IP included activities and guidelines, which could be used at various levels, for improving MDA coverage within the community

## Results (comparison of baseline and endline)

### Profiling the respondents of the households

**Fig 1:**
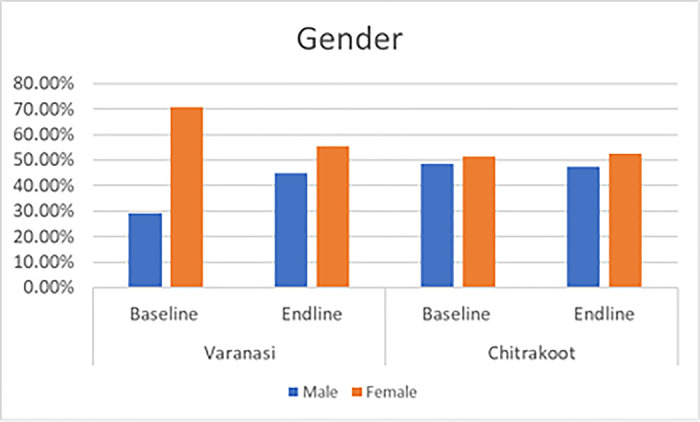
% of participants as per Gender.

**Fig 2:**
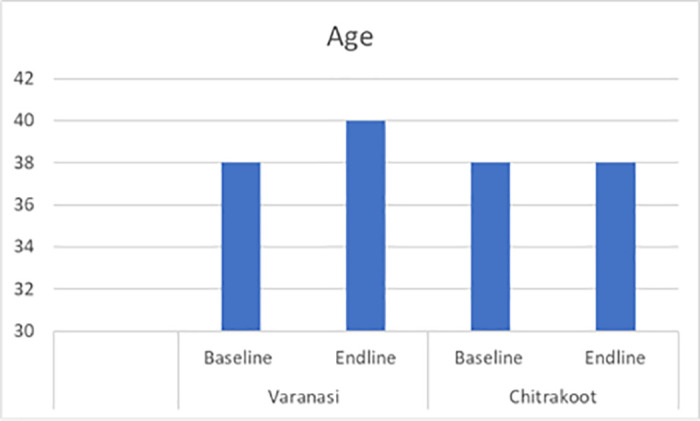
% of participants as per Age.

**Fig 3:**
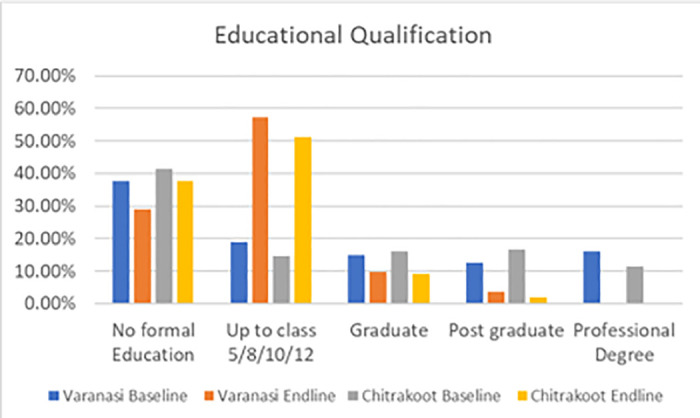
% of participants as per Educational Qualification.

**Fig 4:**
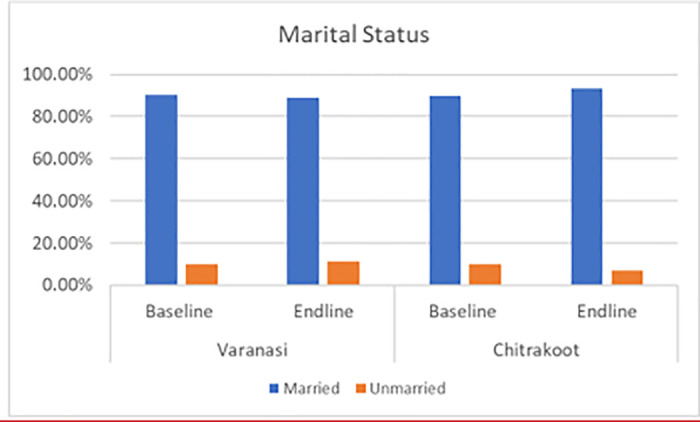
% of participants as per Marital Status.

**Fig 5:**
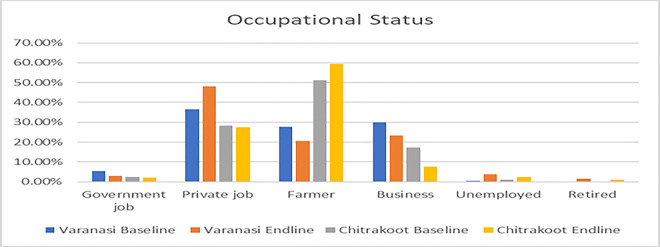
Occupational Status of the respondants.

**Fig 6:**
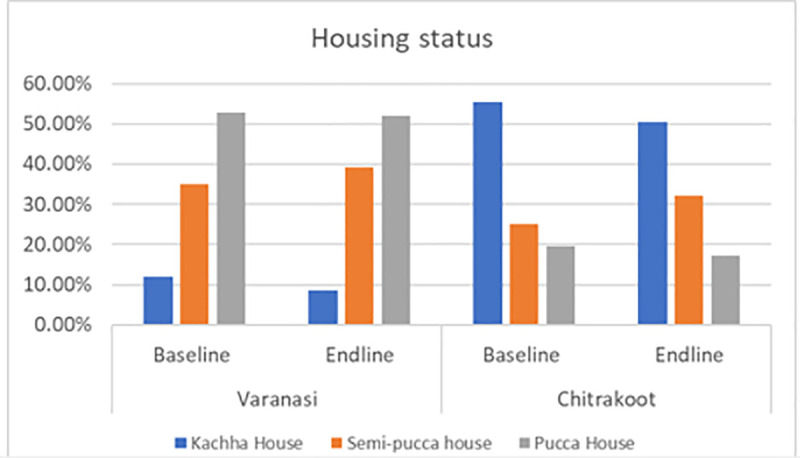
Housing Status of the respondants.

**Fig 7:**
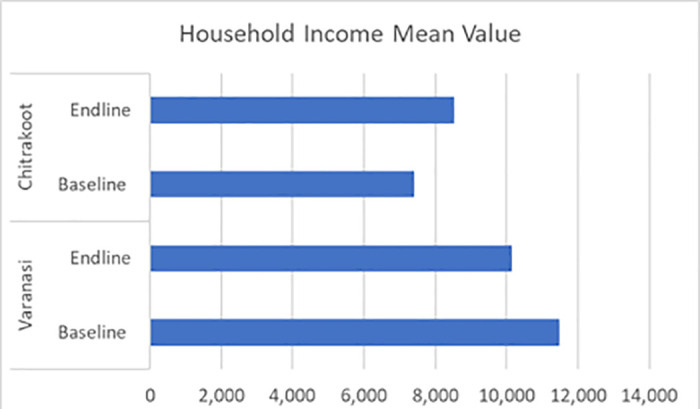
Household income (in INR).

The following charts present the various indicators of the socio-demographic and economic profiles of the households covered under the quantitative and qualitative components of the study during the pre- and post-intervention survey. The mean age of the respondents from both the study districts was 38 years. Most of the respondents were female, which was reported to be approximately seven out of ten in Varanasi and a little over five out of ten in Chitrakoot. The team of ethnographers reported that a relatively lower proportion of responses from women were captured in Chitrakoot due to the deep-rooted patriarchal structure, which prevented the female members of the family from talking to strangers. In instances where the women communicated with the investigators, the conversation occurred under the watchful eyes of a male member of the family. A sizeable proportion (around four out of every ten) had no formal education in both the study districts. In Varanasi, respondents reported that their primary occupation was a private job (37%), followed by business (30%), whereas the respondents in Chitrakoot reported that farming (51%), followed by private jobs (29%), were the main occupations. The dwelling units in Chitrakoot were mostly *kachha (mud wall with thatched roof)*. The mean monthly household incomes in Varanasi and Chitrakoot were reported to be around Indian rupees (INR) 11,500 and INR 7,500 respectively. To conclude, the respondents covered in the study were mostly young, less educated, married, and economically poor. Figs 1–7 provides the demography of the respondents. Fig 1 provides the % of participants as per Gender. Fig 2 provides the % of participants as per Age. Fig 3 provides the % of participants as per Education qualification. Fig 4 provides the marital status of Respondents. Fig 5 describes the occupational status of the respondents. Similarly, Figs 6 and 7 describes the housing status and household mean income of the respondents.

### Rapid ethnography

The study team conducted 24 participatory mapping exercises, 231 key informant interviews, 175 case interviews (68 with people who took LF drugs in 2019 and 107 with those who did not), 204 mini-interviews during 48 transect walks, and 1,123 short surveys. The table below shows various kinds of data collection methods, together with their numbers, that were conducted in rapid ethnography (Table 4).

**Table 4:** Data collection of rapid ethnography.

S. No.	District	Village	Participatory mapping	Transect walks	Key informant interview	Case interview (compliant)	Case interview (non-compliant)	Mini interview	Short survey	TOTAL
1	Varanasi	Kashi Vidyapeeth	4	8	40	21	17	42	161	281
2		Harhua	4	8	46	14	7	32	210	309
3		Shivpur	4	8	41	13	14	43	218	329
4	Chitrakoot	Pahari Bujurg	4	8	38	12	12	25	176	263
5		Shivrampur	4	8	28	1	33	22	185	269
6		Karwi	4	8	38	7	24	40	173	282
Total target set			24	48	240	60	60	120	600	1,080
Total target achieved			24	48	231	68	107	204	1,123	1,733

In the rapid ethnography, people were asked about their knowledge of the disease and how it spreads. This helped with understanding the necessary precautions that people opt for against LF disease. Respondents in Varanasi reported varied perceptions on transmission of LF. In Chitrakoot, while the community leaders and influencers were aware of the disease, however, they did not have knowledge related to the cause and prevention of the disease. Some of the respondents could not explain what causes the disease and how it spreads.

A lot of myths prevailed around this disease in the community with respect to the related causes and spread of the disease. These Myths are provided in the Table 5.

**Table 5:** Myths related to causes of LF.

It is a genetic disease. This disease is caused by blood deficiency as well as lack of proper diet. LF spreads by coming in contact with the infected person. LF is caused because of any insect bite or because of any old injury or wound. It is a disease that happens if it is destined to. LF is a result of past life deeds

The major reasons that came out as the major barriers of successful MDA coverage during the rapid ethnography are given in the Table 6.

**Table 6:** Major barriers to successful MDA.

Varanasi	Chitrakoot
Community’s perception Could not understand the precautionary nature of medicines Lack of awareness around transmission of LF Aversion towards allopathic medicines Fear of ADR, number of tablets & uncovered tablets Lack of trust on ASHAs; ASHAs practice class- based discrimination Lack of awareness towards MDA	Community’s Perception Lack of awareness around MDA and LF Uncovered tablets Could not understand the precautionary nature of medicines Dependence on govt. for health care seen as a sign of helplessness ASHA workers discriminate on the basis of caste Fear of ADR
Drug administrators’ perception Overworked, underpaid and delayed payments Working male members not available at home; some refuse to take medicine from a woman Demeaned by “upper class” people; Some think the medicines cause infertility Lack of coordination between health workers and ER Exhaustion of tablets (increased population) Short notice (no time for pre-MDA survey)	Drug administrators’ perception Overworked, underpaid and delayed payments Seasonal migration Demeaned by “upper” caste people Educated v/s uneducated Exhaustion of tablets (Increased population) Male members not available at home

### Awareness and perception regarding MDA

#### Percentage of informed HHs.

**Fig 8:**
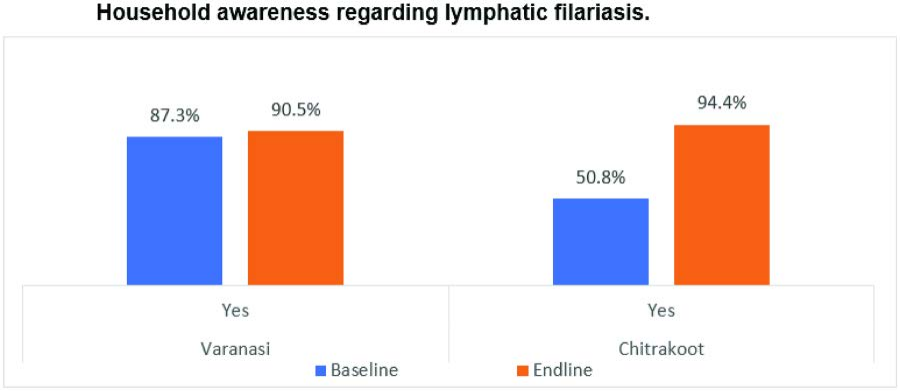
Household awareness regarding lymphatic filariasis.

In the HH quantitative survey, the respondents were asked about their knowledge of LF. In both study districts, awareness regarding LF increased in the post-implementation phase (Fig 8). Chitrakoot community awareness about the disease has gone up from 50.8% of HHs at the baseline survey to 94.4% at the end-line survey, a statistically significant change (CI = 95%’ χ2 = 314.93, p < 0.001). Even in Varanasi, an increase of about 3 percentage points was observed in the awareness level of respondents, also statistically significant (CI = 95%’ χ2 = 3.43, p < 0.05).

This increase in awareness regarding LF was also reflected in IDIs with the community leaders and influencers, wherein, contrary to the baseline, almost all the respondents had heard about the disease and MDA.

During our field team’s interaction with the community leaders and influencers in Varanasi, it was brought to light that only some of them knew that there was a cure for the disease. The majority of the people cited having no knowledge and information pertaining to LF’s cure. Box 1 below gives a verbatim quote by a community influencer regarding the perception of how the disease can be controlled. Similarly, Box 2 gives a quote, by another community influencer on how the disease can by treated

**Box 1. Quote by a community influencer**

It can be controlled by proper medication and sanitation facilities nearby the residence.

- Community influencer, Nasirpur, Kashi Vidyapeeth, Varanasi

**Box 2. Quote by a community influencer**

It can be treated by taking allopathic medicines

- Community influencer, Ram Nagar, Koh, Chitrakoot

#### Sources of information.

In the HH quantitative survey, all the respondents who knew about LF were further inquired about the source of this information. Health workers/ Accredited Social Health Activists (ASHAs) were the primary source of information in both Varanasi (46.2% of HHs) and Chitrakoot (53.2% of HHs) in the post-implementation phase (Table 7). In addition, word of mouth via family, friends, and neighbors was found to be the second most common source of information in both study districts. Apart from this, mass media (such as TV, radio, posters, banners, etc.) served as the source of information for a minuscule proportion of the respondents.

**Table 7:** Sources of information regarding lymphatic filariasis, per district and phase.

Intervention	Varanasi		Chitrakoot		Overall	
	Pre (n = 579)	Post (n = 593)	Pre (n = 334)	Post (n = 622)	Pre (n = 913)	Post (n = 1,215)
TV	6.3%	5.2%	16.2%	2.4%	8.5%	3.8%
Newspaper	2.3%	1.5%	2.2%	0.3%	2.3%	0.9%
Hoarding/ Poster/ Banner	1.8%	1.2%	2.2%	0.3%	1.9%	0.7%
Public announcement	0.5%	3.0%	7.6%	0.2%	2.0%	1.6%
Health worker	51.6%	46.2%	36.8%	53.2%	48.3%	49.8%
Relatives	10.0%	12.3%	16.2%	9.3%	11.4%	10.8%
Neighbors/ friends	27.5%	30.5%	18.4%	34.2%	25.5%	32.4%

#### HHs visited for drug administration (reach of DAs).

**Fig 9:**
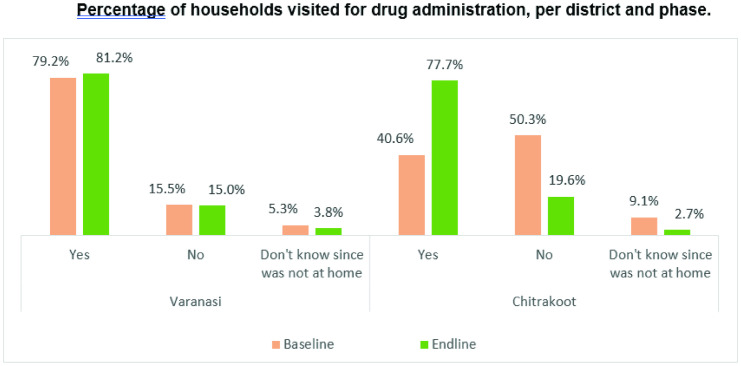
Percentage of households visited for drug administration, per district and phase.

All the respondents were asked whether any DA visited their HH to provide them with the LF drugs. While in Varanasi the proportion reportedly increased by 2 percentage points in the MDA round post-implementation of the intervention measures, the same almost doubled in Chitrakoot, from 40.6% to 77.7% at the time of the end-line survey (Fig 9). This was found to be a statistically significant increase (CI = 95%, χ^2^ = 164.46, p < 0.001).

In Chitrakoot, it was found that some hamlets of the village received medicines during the MDA rounds whereas rest of the hamlets did not. A health official pointed this out and his verbatim quote has been given in Box 3. Another community infuencer pointed out the reason why few houses are missed, the quote mentioned in Box 4.

**Box 3. Quoted by a health official**

ASHAs are not able to convince families in their respective villages about the benefits of consuming LF drugs; therefore, people are not inclined to take them

- Health official, Chitrakoot

**Box 4. Quoted by a community influencer**

Houses that are not located near the main village are often left.

- Community influencer, Pahi village, Karvy block, Chitrakoot district

#### Drug coverage (individuals).

**Fig 10:**
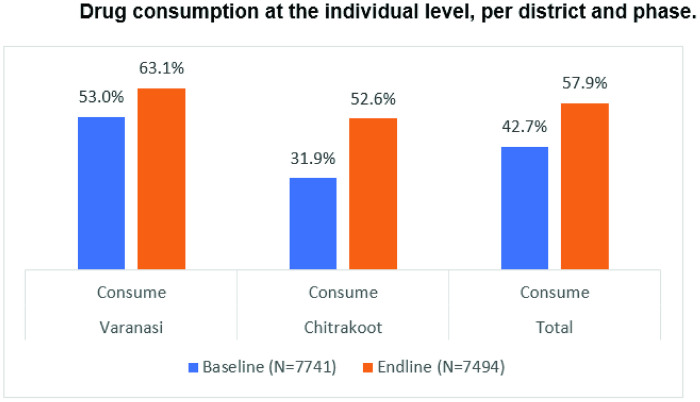
Drug consumption at the individual level, per district and phase.

The study also analyzed drug consumption at the individual level. Out of 7,741 and 7,494 individuals in the baseline and end-line surveys, respectively, consumption of drugs during MDA rounds increased in both study districts at the time of the end-line assessment compared to baseline: in Varanasi, even though statistically not significant, consumption increased from 53.0% to 63.1%; and in Chitrakoot, a statistically significant increase (CI = 95%’ χ^2^ = 9.023, p < 0.001) from 31.9% to 52.6% was reported (Fig 10).

**Fig 11:**
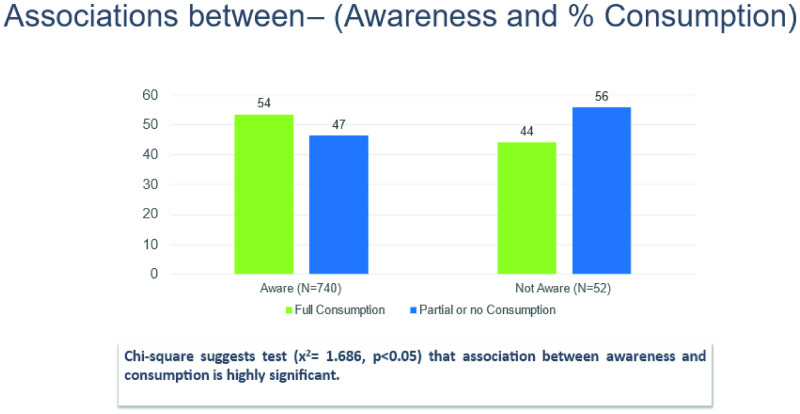
Associations between Awareness and % consumption.

Even though the coverage in Chitrakoot was only 52.6% in the post-intervention MDA campaign, there was a significant improving in the coverage (>20%) in comparison to the previous MDA round. This was due to an increase in awareness as well as increase in the visit of the DAs to the respective communities during the MDA campaign. There is a significant association between awareness and consumption of drugs which was evident during the study. Chi-square suggests test (x^2^ = 1.686, p < 0.05) that association between awareness and consumption is highly significant. Chi-square suggests statistical significance (x^2^ = 29.338, p-value<0.05) between (Interpersonal communication) IPC Awareness and LF drug consumption. Fig 11 represents the above-mentioned association where full consumption means all the members of the HH had consumed the drugs whereas partial or no consumption means that either some or no one in the HH consumed all the drugs

### Reasons for nonconsumption of drugs

#### Quantitative.

The individuals who did not consume the LF drugs during the MDA round were further questioned about the reasons for this non-consumption through administration of a structured quantitative tool. Some of the major reasons given for non-consumption of drugs are listed in Table 8. Similar themes also emerged from the IDIs with various stakeholders. An field worker pointed out the main reason according to her of people abstaining from drug consumption. Her quote has been included in Box 5.

**Table 8:** Household reasons for nonconsumption of drugs (quantitative analysis).

Reasons	Varanasi		Chitrakoot		Overall	
	Pre (n = 885)	Post (n = 564)	Pre (n = 329)	Post (n = 688)	Pre (n = 1,214)	Post (n = 1,252)
Not at home/work	23.28%	21.45%	16.41%	52.47%	21.42%	38.50%
Fear of side effects or adverse reactions	39.32%	37.41%	40.43%	14.53%	39.62%	24.84%
On treatment for a chronic illness	12.99%	20.92%	2.74%	5.96%	10.21%	12.70%
Not at home/school	10.51%	6.74%	9.12%	13.81%	10.13%	10.62%
Lack of information about mass drug administration	3.50%	6.03%	4.86%	4.36%	3.87%	5.11%
Healthy status (do not need drugs)	8.81%	3.90%	22.19%	6.25%	12.44%	5.19%
Empty stomach	0.68%	1.60%	0.91%	1.74%	0.74%	1.68%
Not at home/college	0.90%	1.95%	3.34%	0.87%	1.57%	1.36%

**Box 5. Quoted by a community influencer**

ADR is the biggest reason for noncompliance. Twenty-five percent of people suffer from ADRs, and the previous MDA saw an increment [incremental increase] in this. PHCs provide medicines to fight ADRs, but it is not enough as the bad word for MDA spreads rapidly.

ASHA worker, Varanasi- Community influencer, Pahi village, Karvy block, Chitrakoot district

#### Qualitative.

Interactions with health officials, DAs, and community leaders and influencers through IDIs and rapid ethnography surveys brought new insight into noncompliance of MDA drugs in the community, from both demand- and supply-side perspectives (Table 9).

**Table 9:** Demand- and supply-side reasons for nonconsumption of drugs (qualitative analysis).

Demand Side	Supply Side
Lack of awareness Unpacked and/or high number of tablets Mistrust in government medicines Fear of adverse drug reactions Lack of understanding around precautionary nature of the drugs Discrimination by drug administrators on the basis of class/caste	Short time for preparedness Poor motivation of drug administrators Exhaustion of medicine tablets in the field Poor quality of family survey Refusal to take from nonlocal female drug administrator Refusal by rich and educated class

From the community (demand) side, the main reasons for noncompliance are lack of awareness regarding LF and MDA, mistrust in government medicines, migration, unpacked medicine tablets and/or high number of tablets for consumption (eight in total), fear of adverse drug reactions, lack of understanding around the precautionary nature of the drugs, and caste/class discrimination by DAs.

From the supply side, main reasons given were the short time provided for MDA preparedness; the community’s reluctance to take drugs from non-local female DAs; exhaustion of tablets in the field; poor quality of family survey leading to an inaccurate target; refusal by the rich and educated to take the medicine, and poor motivation of DAs due to delayed payments and high workload.

### Motivational score of DAs

Motivation of DAs was gauged based on their perceptions and using a set of 20 statements across components such as support and recognition from community and supervisors and self-motivation. The main reasons for an increase in motivational scores were:

-  Receipt of past honorarium.
-  Appreciation by supervisors.
-  Decrease in the daily workload.
-  Good-quality training.

To measure the level of change in their motivation post-intervention, a total motivation score was calculated (Table 10) for both baseline and end-line (N = 60 each). Due to non-normality of the variable and since the variable is not an ordinal one, a Mann Whitney U test was carried out. The end-line has a higher mean rank (79.58) than the baseline (41.42) and thus tends to take higher values. A statistically significant difference was found between end-line and baseline for the total motivation score (U = 655.00, p < .001), thereby indicating a statistically significant improvement in terms of motivation among all the DAs at the time of end-line as compared to baseline.

**Table 10:** Motivational score of drug administrators (DAs) for mass drug administration (MDA).

S. No.	Questions	Total (N)	Pre (N)	Mean rank	Sum of ranks	Post (N)	Mean rank	Sum of ranks
Q34.1	My role as a DA is important to the success of the MDA program - Options	120	60	44.88	2,693.0	60	76.12	4,567.0
Q34.2	I have clear goals that I work towards during MDA - Options	120	60	45.25	2,715.0	60	75.75	4,545.0
Q34.3	I feel confident when I carry out MDA program activities - Options	120	60	55.45	3,327.0	60	65.55	3,933.0
Q34.4	I feel I have the tools and job aids required to engage with the community before and during MDA - Options	120	60	54.70	3,282.0	60	66.30	3,978.0
Q34.5	I am satisfied with the training I received to perform my job as a DA - Options	120	60	48.09	2,885.5	60	72.91	4,374.5
Q34.6	I am happy with the honorarium that the MDA program provides me for my pocket expenses - Options	120	60	58.58	3,515.0	60	62.42	3,745.0
Q34.7	I feel appreciated and supported by the MDA program - Options	120	60	56.96	3,417.5	60	64.04	3,842.5
Q34.8	My involvement as DA in MDAs has improved in community awareness on health services – Options	120	60	55.08	3,305.0	60	65.92	3,955.0
Q34.9	I think that the drugs I give to the community are effective - Options	120	60	54.70	3,282.0	60	66.30	3,978.0
Q34.10	I am motivated by the positive attitude of the community towards me - Options	120	60	55.44	3,326.5	60	65.56	3,933.5
Q34.11	To be effective, no extra support is required from the MDA program - Options	120	60	57.00	3,420.0	60	64.00	3,840.0
Q34.12	I feel supported and appreciated by the community - Options	120	60	54.79	3,287.5	60	66.21	3,972.5
Q34.13	I get supportive feedback from my supervisor during MDA to improve my work - Options	120	60	53.16	3,189.5	60	67.84	4,070.5
Q34.14	My good performance is recognized by my supervisor – Options	120	60	50.45	3,027.0	60	70.55	4,233.0
Q34.15	I believe that the community follows my advice on the importance of taking the medicines - Options	120	60	58.06	3,483.5	60	62.94	3,776.5
Q34.16	I feel safe when I go door-to-door to distribute medicines - Options	120	60	45.64	2,738.5	60	75.36	4,521.5
Q34.17	The community thinks that the medicines are safe and effective - Options	120	60	55.88	3,352.5	60	65.13	3,907.5
Q34.18	My responsibilities as a DA do not interfere with my responsibilities at home or at my primary job - Options	120	60	59.63	3,577.5	60	61.38	3,682.5
Q34.19	I do not feel stressed and emotionally drained at the end of each MDA day - Options	120	60	50.96	3,057.5	60	70.04	4,202.5
Q34.20	I know what to do if there are adverse drug reactions during MDA - Options	120	60	54.73	3,283.5	60	66.28	3,976.5

### Intervention package

The following intervention package (Table 11) was developed based on the analysis of the data of the pre-intervention phase. The Government agreed that all interventions were essential for improvement in coverage but due to lack of resources and the ongoing pandemic it was not possible to implement every recommended intervention.

**Table 11:** The intervention Package.

Level	Interventions
**National**	Procurement and supply of MDA drugs to state at least 3 months in advance. Procure blister packed MDA drugs as far as plausible to enhance drug compliance in the community. Having a celebrity as LF/MDA Ambassador
**State**	Dates of MDA to be Notified to districts at least 2 months in advance. Provision and timely release of funds with relevant guidelines to districts/blocks for IEC material and payment of honorarium to DAs MDA preparedness of the district be assessed by state level officer/partners, using standardized preparedness assessment checklist, starting two weeks before MDAs.
**District**	GANT chart with timeline of key activities leading to MDA start date in each district must be prepared in advance and adhered to. Training of Block level Master trainers 5–6 weeks prior to MDA date. Participants should include Block Community Process Managers (BPCM) also. District Task Force (DTF) must be in place and should be convened at least thrice:
	First, at the start of preparatory phase, one month prior to MDA date. Second, to assess the preparedness. Third, to review the MDA campaign at the end of the activity.
	Media Briefing on MDA, benefit of MDA drug consumption, ADR specific risk communication & its management should be conducted 1 week of MDA campaign. Identity card for all Drug Administrators (DAs) to be issued by CMO office.
**Block**	Training of DAs: Training of DAs to be completed 1 month prior to MDA date. The number of participants per batch for Training should be < 50. Training of DAs should be done as per standardized training curriculum and using training module. One Angadwadi Worker (AWW)/ volunteer from each village (preferably woman) to be identified to support the DA in community. They should be trained along with DA. Family survey: After the training, the DA should complete the family survey by visiting each family in her area/village, and using this opportunity for IPC to reinforce MDA date and importance of drug compliance. Micro planning - On completion of the family survey, microplanning covering all Households (HH) of the villages, small hamlets, hard to reach areas and high-risk population/groups like (migratory/ brickkilns/ factory workers/ construction site/ rag pickers) etc. to be completed 2 weeks prior to MDA and submitted by Block MO to the District for review. An information kit for DAs to be developed for use in the community during IPC. Adverse Drug Reaction (ADR) management protocol must be in place at block level and ADR management kit to be made available with all supervisors. Constitution of RRT at planning unit should be done and this RRT should be functional to respond to any ADRs reported in the field during MDA campaign.
**Village/ Community**	MDA campaign to be launched/inaugurated by village Leader/ Pradhan/Sarpanch/MLC/MLA/PRI representative. DA to revisit the missed HH in the evening to cover/administer drug to left-outs/ missed members during day. Enlist those houses that are partially covered/Locked house/Resistance house/Family out of village and prepare a second re-visit plan to cover the left outs. Establish a drug sub-depo/center in the village either at ASHA’s house/AWC/HSC for anyone to come and consume drugs in the village beyond working hours throughout the MDA campaign duration
**Partners Level:** All partners should make a comprehensive monitoring plan of the preparatory phase and MDA campaign and submit it to the state programme officer.	
**Documents developed:** MDA preparedness standardized checklist Modified/revised Standardized Training curriculum for DAs with agenda and training methods. ADR management kit and protocol for DAs/Supervisors	

Due to ongoing COVID19 and many operational issues, Govt. of Uttar Pradesh the government of UP implemented the following during post-implementation phases during subsequent MDA campaign in the study districts.:

- Notifying date of MDA well in advance,
- Provision and timely release of funds and logistics with relevant guidelines to districts/blocks for IEC material and payment of honorarium to DAs,
- Training of block level master trainers 5-6 weeks before MDA,
- District Task Force under chairmanship of district magistrate review the preparedness and implementation of MDA campaign,
- Media briefing on MDA 1wwek before the campaign,
- Training of DAs 1 month prior to the campaign using standardized training curriculum and training module
- Completion of household survey by DA after visiting every family and using this opportunity for IPC benefit of drug consumption
- Microplanning extensively covering all Households (HH) of the villages, small hamlets, hard to reach areas and high-risk population/groups like (migratory/ brick-kilns/ factory workers/ construction site/ rag pickers)
- Launching of MDA campaign launched by village leader/ PR representatives,
- Revisit the missed HH in the evening to cover/administer drug to left outs/ missed members during day by the DA.
- Providing I-cards to MDA team in the urban areas

The study team developed the following for utilization by the Government during the subsequent MD campaign:

-  MDA preparedness standardized checklist
-  Modified/revised Standardized Training curriculum for DAs with agenda and training methods.
-  ADR management kit and protocol for DAs/Supervisors

## Discussions

### Study takeaways

The aim of the study was to generate evidence to inform the India country program on improving future MDA results through identifying factors that are critical for achievement of high coverage during MDA campaigns.

As per WHO recommendations, five annual rounds of MDAs with optimal coverage of 65% and above is sufficient to stop LF transmission. UP has conducted more than ten rounds of MDAs, but still 50 of the 51 districts have not cleared TAS. Results from the study suggest that there are multiple drivers that impede true community drug consumption, resulting in poor coverage. The two study sites (Varanasi and Chitrakoot Districts in UP) are both TAS-failed districts. The overall results indicate that, with interventions, there was improvement in the knowledge about, attitude toward, and practices around LF and MDA by respondents, as well as in coverage. Also, it was observed that there was commitment by UP leadership (state program officer) to use/apply the IP developed after analyzing data from the baseline study (pre-intervention phase).

The baseline data (data from previous MDAs collected through quantitative and qualitative methods) revealed that the main source of information on LF and MDA was from health workers/ASHAs. Awareness about LF and MDA was poor, as Chitrakoot community leaders and influencers reported no knowledge about the MDA round, and at baseline the percentage of houses visited by DAs during the MDA was only 40.6% and 79.2% in Chitrakoot and Varanasi, respectively. Despite Chitrakoot’s low coverage, the district had a higher consumption rate, with approximately 70% reporting every single member of the HH consuming the MDA drugs.

HHs that belonged to lower economic strata demonstrated higher consumption of drugs as compared to affluent HHs. Additionally, community members, including village leaders, actively supported study activities and participated in the IDIs and HH surveys. The involvement of the community leaders and influencers helped in creating awareness in the community and encouraging better participation and acceptance. Thus, active involvement by local leaders/influencers proved to be a powerful enabling factor in the success of a large-scale campaign like MDA. Another facilitating factor was trust in the health workers: where people held high regard for the efforts put in by the health workers, they consumed the tablets provided to them during MDA.

One of the other main reasons of non-consumption was the fear of side effects of the drugs among the community. This was mainly due to lack of awareness about Lymphatic Filariasis in general and MDA campaigns in particular. This result was congruent to the study conducted in 4 endemic districts in UP before [6]. The study was conducted across Amethi, Lucknow, Raebareli, and Sultanpur, analyzing coverage, compliance, and factors influencing effective MDA coverage.)In some study geographies, the reason of non-consumption was lack of visits to the households by the DAs. This was also evident in a previous study conducted in Prayagraj district in Uttar Pradesh [7].

The IP was used by the government of UP during the pre-MDA preparatory phase for subsequent MDA campaign planning and implementation. The Government’s commitment to improving coverage was demonstrated during the MDA planning process when the state program officer notified districts of the MDA date six weeks in advance, which helped the districts and implementation units in timely planning of the preparatory activities, like training-of-trainers (ToT), DA trainings using the DA training module developed through the IP, micro-planning, family survey, timely supply of drugs, handling of logistics, and provision of information, education, and communication (IEC) materials, etc.

Overall consumption of drugs increased in both study districts between the baseline and end-line assessments. Compared to baseline, even though statistically not significant, consumption of LF drugs during MDA rounds increased from 53.0% to 63.1% in Varanasi. In Chitrakoot, a statistically significant increase (CI = 95%’ χ^2^ = 9.023, p < 0.001) in consumption of drugs, from 31.9% to 52.6%, was reported.

During both the baseline and end-line surveys, it was observed that a greater proportion of women consumed the drugs as compared to men. This difference was more pronounced in Chitrakoot District, wherein 57.3% of women surveyed consumed LF drugs during the MDA round as compared to 48.4% of men (during the end-line survey). This difference could be attributed to male members of the family not being at home at the time of the DA’s visit, as they normally leave home for work.

The study showed that awareness and understanding of LF increased in the post-implementation phase. In Chitrakoot there was an increase in community awareness from 50.8% to 94.4%, or about 44 percentage points, post-intervention. The dissemination of information on public health interventions is very critical to enhancing public acceptability. Moreover, in the community there are a lot of myths regarding the causes and spread of LF, such as LF is a genetic disease, is caused by blood deficiency, spreads through contact with an infected person, is caused by insect bites or old injuries, and is the result of past life deeds. Therefore, community awareness about disease and MDA needs to be enhanced through interpersonal communication through health workers/ASHAs and electronic media. It should be an ongoing activity, regardless of the date for MDA.

Being afraid of side effects and being away from home during the DA’s visit were found to be the main reasons for noncompliance with drug consumption. Therefore, there is a need for strengthening IEC activities in the community and having DAs revisit HHs in the evening or early the next morning to cover any missed people to improve drug consumption.

### Barriers to high MDA coverage

A range of social, behavioral, and systemic factors were identified as barriers to achieving high MDA coverage:

1. **Misinformation and Misconceptions:** Many community members harbored misconceptions regarding the MDA drugs, including concerns about side effects and doubts about the necessity of the treatment. This reluctance was particularly evident in populations with lower literacy levels.
2. **Community Trust Issues:** Some participants expressed a lack of trust in health workers and the government, leading to refusals in drug consumption. Mistrust was exacerbated by past experiences with government health programs.
3. **Access Challenges:** Geographical barriers, particularly in Chitrakoot, made access to MDA campaigns difficult. The lack of adequate outreach efforts in remote areas further contributed to lower coverage.
4. **Operational Gaps:** Issues such as inadequate training of front-line health workers, insufficient supervision, and lack of motivation among drug distributors affected the efficiency of drug delivery and uptake.
5. **Household-Level Influences:** The study found that decisions regarding MDA drug consumption were often made at the household level. If the head of the household was skeptical, other members were less likely to participate.

### Enablers for improved coverage

Despite these barriers, several enabling factors contributed to the improvements observed:

1. **Community Engagement Strategies:** Interventions that included interactive community awareness sessions and direct engagement by local influencers (such as religious leaders and community heads) significantly increased acceptance.
2. **Enhanced IEC Campaigns:** Strengthening information, education, and communication (IEC) campaigns with localized messaging and vernacular language materials helped address misinformation and encouraged drug acceptance.
3. **Support from Health Workers and Volunteers:** Training and equipping frontline health workers with better communication tools and motivation strategies improved their effectiveness in persuading communities to participate.
4. **Leveraging Technology:** The use of mobile messaging and digital reminders helped increase awareness and compliance rates among younger and tech-savvy demographics.
5. **Political and Administrative Will:** The commitment from state-level authorities to use study insights for programmatic improvements indicates a conducive policy environment for better MDA execution in the future.

### Implications for policy and practice

The findings of this study have significant implications for the design and implementation of future MDA programs. Addressing misconceptions, strengthening community engagement, and ensuring operational efficiency must be at the core of the intervention strategies. Policy recommendations include:

-  Developing a **community-centered approach** that involves local influencers and trusted figures in promoting MDA.
-  Improving **logistical planning** to ensure that hard-to-reach populations are adequately covered.
-  Strengthening **training and incentives** for frontline health workers to enhance their outreach effectiveness.
-  Utilizing **data-driven decision-making** by leveraging monitoring and evaluation systems to refine MDA strategies in real time.

### Study limitations

The COVID-19 pandemic posed multiple challenges for the study team, grossly affecting the study timeline, community interaction, and data collection. Most of the collaboration/meetings with the government and partners were done virtually, and following the advice of the ethical committee, focus group discussions for community influencers and leaders were changed to IDIs. Additionally, simultaneous MDA campaigns in Varanasi and Chitrakoot were not conducted because of a government decision around the pandemic, leading to twice the effort in data collection in the field. Finally, though the quality of supervision improved, the number of supervisors decreased due to the involvement of most of the supervisors (including auxiliary nurse midwives) in the COVID-19 vaccination drive.

The participants of the study were randomized, with inclusion of both genders of people over 18 years old. Due to ethical regulations, no information on caste or religious affiliation was collected. The HHs were randomized and there was no exclusivity in the study.

## Conclusion

Observations from this study showed that, generally, community members’ knowledge of, attitudes toward, and practices around MDA improved from the pre- to the post-intervention phase. Many factors have been proven to be important in implementing and improving coverage of MDA. Significant among these are timely notification by leadership of the MDA dates (eight weeks in advance), timely planning, adequate funding, addition of IEC materials, high-quality interactive training of DAs, involvement of community leaders and influencers, better coordination, and partnership, and dedicated daily review mechanisms at the district level.

While UP has faced persistent challenges in eliminating LF despite multiple MDA rounds, the study highlights key opportunities for improvement. By addressing the identified barriers through targeted interventions and leveraging enabling factors, there is potential to significantly enhance MDA effectiveness. Future efforts should prioritize community engagement, trust-building measures, and operational efficiencies to ensure sustained high coverage and successful LF elimination in the region.
